# A novel predictive model using routinely clinical parameters to predict liver fibrosis in patients with chronic hepatitis B

**DOI:** 10.18632/oncotarget.19501

**Published:** 2017-07-22

**Authors:** Jian Wang, Xiaomin Yan, Yue Yang, Haiyan Chang, Bei Jia, Xiang-An Zhao, Guangmei Chen, Juan Xia, Yong Liu, Yuxin Chen, Guiyang Wang, Li Wang, Zhaoping Zhang, Weimao Ding, Rui Huang, Chao Wu

**Affiliations:** ^1^ Department of Infectious Diseases, Nanjing Drum Tower Hospital, Clinical College of Nanjing Medical University, Nanjing, Jiangsu, China; ^2^ Department of Infectious Diseases, Nanjing Drum Tower Hospital, Nanjing University Medical School, Nanjing, Jiangsu, China; ^3^ Department of Infectious Diseases, Nanjing Drum Tower Hospital, Clinical College of Traditional Chinese and Western Medicine, Nanjing University of Chinese Medicine, Nanjing, Jiangsu, China; ^4^ Department of Infectious Diseases, Affiliated Hospital of Nanjing, University of Traditional Chinese Medicine, Nanjing, Jiangsu, China; ^5^ Department of Laboratory Medicine, Nanjing Drum Tower Hospital, Nanjing University Medical School, Nanjing, Jiangsu, China; ^6^ Department of Hepatology, Huai’an No. 4 People's Hospital, Huai’an, Jiangsu, China

**Keywords:** chronic hepatitis B, liver biopsy, liver fibrosis, serum biomarkers, non-invasive fibrosis model

## Abstract

**Objectives:**

Noninvasive models have been established for the assessment of liver fibrosis in patients with chronic hepatitis B(CHB). However, the predictive performance of these established models remains inconclusive. We aimed to develop a novel predictive model for liver fibrosis in CHB based on routinely clinical parameters.

**Results:**

Platelets(PLT), the standard deviation of red blood cell distribution width(RDW-SD), alkaline phosphatase(ALP) and globulin were independent predictors of significant fibrosis by multivariable analysis. Based on these parameters, a new predictive model namely APRG(ALP/PLT/RDW-SD/globulin) was proposed. The areas under the receiver-operating characteristic curves(AUROCs) of APRG index in predicting significant fibrosis(≥F2), advanced fibrosis(≥F3) and liver cirrhosis(≥F4) were 0.757(95%CI 0.699 to 0.816), 0.763(95%CI 0.711 to 0.816) and 0.781(95%CI 0.728 to 0.835), respectively. The AUROCs of the APRG were significantly higher than that of aspartate transaminase(AST) to PLT ratio index(APRI), RDW to PLT ratio(RPR) and AST to alanine aminotransferase ratio(AAR) to predict significant fibrosis, advanced fibrosis and cirrhosis. The AUROCs of the APRG were also significantly higher than fibrosis-4 score (FIB-4) (0.723, 95%CI 0.663 to 0.783) for cirrhosis(P=0.034) and better than gamma-glutamyl transpeptidase(GGT) to PLT ratio(GPR) (0.657, 95%CI 0.590 to 0.724) for significant fibrosis(P=0.001).

**Materials and Methods:**

308 CHB patients who underwent liver biopsy were enrolled. The diagnostic values of the APRG for liver fibrosis with other noninvasive models were compared.

**Conclusions:**

The APRG has a better diagnostic value than conventionally predictive models to assess liver fibrosis in CHB patients. The application of APRG may reduce the need for liver biopsy in CHB patients in clinical practice.

## INTRODUCTION

Hepatitis B virus (HBV) infection is a serious public health problem globally. Liver fibrosis and cirrhosis are major reasons of morbidity and mortality in patients with chronic hepatitis B (CHB) [1]. Assessing the stages of liver fibrosis in CHB patients could help clinicians predict the disease progression and formulate the optimally therapeutic schedule to avoid sever complications [2]. Therefore, it is essential to identify the degree of liver fibrosis to better manage such patients.

Currently, liver biopsy (LB) is recognized as the gold standard for estimating histological stages of liver diseases [3, 4]. However, LB is an invasive and costly procedure which is very difficult to widespread utilization in routine practice. Meanwhile, sampling errors and observer discrepancy associated with liver biopsy may bias the result of liver fibrosis. Moreover, it does not allow the dynamic observation of liver fibrosis by LB. Therefore, non-invasive, inexpensive and convenient methods for assessing liver fibrosis are urgently needed.

Transient elastography (TE) is a promisingly noninvasive method that evaluates liver stiffness and has been reported to accurately assess the degree of liver fibrosis in CHB patients [5–7]. However, the technique is relatively high cost and susceptible by some factors such as necroinflammatory activity, total bilirubin (TBIL), obesity, which may also limit the clinical application [8, 9]. Simply noninvasive fibrosis tests (NITs) including aspartate transaminase (AST) to platelet (PLT) ratio index (APRI) and the fibrosis-4 score (FIB-4) using two or three inexpensive laboratory tests to predict hepatic fibrosis have been studied and validated over the past decade [10–13]. WHO published the guideline on the management of CHB infection in 2015, which recommended the use of APRI as a noninvasive test to assess significant liver fibrosis and cirrhosis in resource-limited regions [8]. Both APRI and FIB-4 were based on patients with chronic hepatitis C (CHC) [11–13], while their value for assessing patients who are chronically infected with HBV remains controversial [14, 15]. Recently, Lemoine et al. reported the gamma-glutamyl transpeptidase (GGT) to PLT ratio (GPR) as a novel and more accurate laboratory marker than classical biomarkers APRI and FIB-4 to assess liver fibrosis in patients with CHB in West Africa populations, but this was not consistent in French cohort [16]. However, Schiavon et al. and Li et al. also demonstrated that GPR did not show better performance in a Brazilian cohort and a Chinese cohort [17, 18]. Other serum-based models, such as AST to alanine aminotransferase (ALT) ratio (AAR), red cell distribution width (RDW) to PLT ratio (RPR) have also been reported to predict significant liver fibrosis and cirrhosis over the past decade in CHB patients, but they are somewhat difficult to use in clinical practice to perform the satisfactory outcomes [19–21]. Therefore, the aim of this study was to establish an improved model based on routinely clinical parameters for the assessment of liver fibrosis in treatment-naïve patients with CHB.

## RESULTS

### Study population

**Figure 1:**
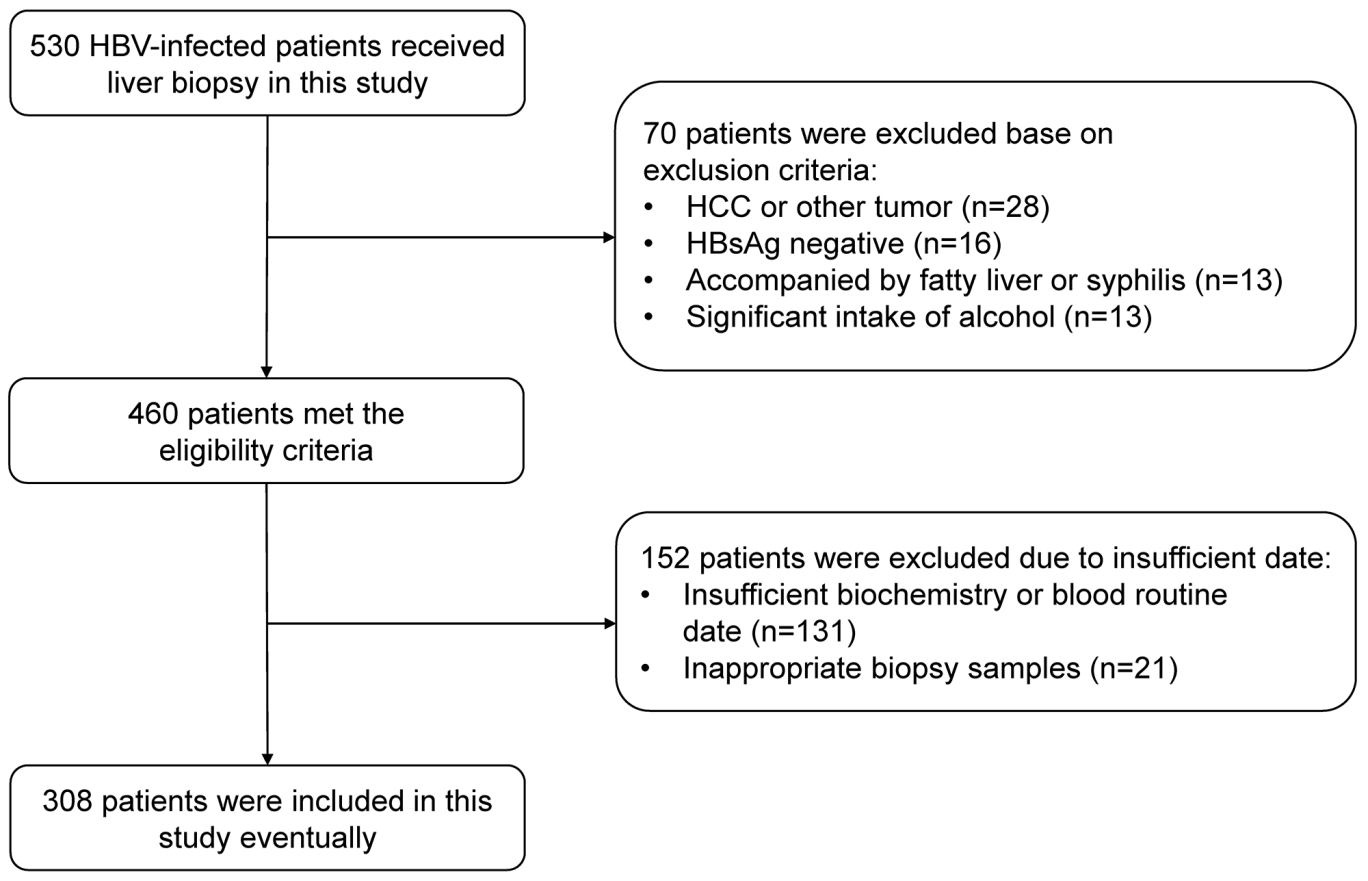
Flow diagram describing the selection of the study population

From January 2008 to December 2016, a total of 530 CHB patients who had undergone a liver biopsy were enrolled in this study. Overall, 70 patients were excluded base on exclusion criteria, 152 patients were excluded due to insufficient data. 308 patients who met the eligibility criteria were included as the final study cohort. Figure 1 presents the flow diagram of the study population. The median (and IQR) age of the CHB patients was 38.5 (29.0-47.0). 165 (53.6%) CHB patients were positive for hepatitis B e antigen (HBeAg) and 230 (74.7%) patients were male. The median (and IQR) ALT and AST level was 40.0 (27.0, 71.0) U/L and 34.0 (25.0, 50.0) U/L, respectively. The distribution of each fibrosis stage in the subjects was as follows: F0, 32 (10.4%) patients; F1, 53 (17.2%) patients; F2, 47 (15.3%) patients; F3, 74 (24.0%) patients; and F4, 102 (33.1%) patients. A detailed demographic and laboratory parameters of the subjects were shown in Table 1.

**Table 1:** Baseline characteristics of the subjects

Characteristics	CHB (n=308)
Median Age (years) (IQR)	38.5 (29.0, 47.0)
Male (%)	230 (74.7)
Median RBC (10^12^/L) (IQR)	4.6 (4.2, 5.0)
Median Neutrophils (10^9^/L) (IQR)	2.8 (2.1, 3.5)
Median Lymphocytes (10^9^/L) (IQR)	1.6 (1.3, 2.0)
Median Hb (g/L) (IQR)	154.0 (145.0, 164.0)
Median RDW-CV (%) (IQR)	12.6 (12.0, 13.5)
Median RDW-SD (fL) (IQR)	46.9 (43.5, 49.3)
Median PLT (10^9^/L) (IQR)	158.0 (118.0, 195.8)
Median TBIL (umol/L) (IQR)	15.2 (11.8, 20.9)
Median Albumin (g/L) (IQR)	45.4 (41.6, 47.5)
Median Globulin (g/L) (IQR)	27.8 (24.4, 31.0)
Median ALT (U/L) (IQR)	40.0 (27.0, 71.0)
Median AST (U/L) (IQR)	34.0 (25.0, 50.0)
Median ALP (U/L) (IQR)	70.0 (59.3, 90.8)
Median GGT (U/L) (IQR)	33.0 (21.0, 65.8)
Median LDH (U/L) (IQR)	174.0 (148.4, 195.0)
Median CHE (U/L) (IQR)	7452.5 (5846.3, 8925.8)
Median TT (s) (IQR)	17.0 (16.3, 17.9)
Median INR (IQR)	1.2 (1.1, 1.2)
HBeAg positive (%)	165 (53.6)
Fibrosis stages	
F0(%)	32 (10.4)
F1(%)	53 (17.2)
F2(%)	47 (15.3)
F3(%)	74 (24.0)
F4(%)	102 (33.1)

RBC: red blood cell; Hb: hemoglobin; RDW-CV: the coefficient of variation of the red blood cell distribution width; RDW-SD: the standard deviation of the red blood cell distribution width; PLT: platelets; TBIL: total bilirubin; ALT: alanine aminotransferase; AST: aspartate aminotransferase; ALP: alkaline phosphatase; GGT: gamma-glutamyl transpeptidase; LDH: lactic dehydrogenase; CHE: cholinesterase; TT: thrombin time; INR: international normalized ratio; HBeAg: Hepatitis B e antigen.

### Construction of a novel model for the assessment of liver fibrosis

In the univariate analysis, the following parameters were identified as positively related to the significant fibrosis for the subjects: age (P<0.001), neutrophilic counts (P=0.024), red blood counts (RBC) (P=0.019), the standard deviation of the red blood cell distribution width (RDW-SD) (P<0.001), PLT (P<0.001), TBIL (P=0.045), globulin (P=0.001), alkaline phosphatase (ALP) (P=0.001), GGT (P=0.04), cholinesterase (CHE) (P=0.008), international normalized ratio (INR) (P=0.019) and thrombin time (TT) (P=0.012). These significant parameters were selected for a multivariate analysis. In the multivariate analysis using the forward stepwise procedures, RDW-SD, PLT, globulin and ALP were the independent predictors of significant fibrosis (Table 2). Finally, using these independent predictors, a new model for predicting liver fibrosis, named the APRG (ALP/PLT/RDW-SD/globulin) index, was derived as follows:

$$\text{APRG index}=\frac{1}{1+e^{-(-6.091+\mathrm{ALP}\times 0.015-\mathrm{PLT}\times 0.01+\mathrm{RDW}-\mathrm{SD}\times 0.118+\mathrm{globulin}\times 0.081)}}$$

**Table 2:** Univariate and multivariate analyses of the relationships between hematological parameters and the significant fibrosis in the entire patients

Variables	Significant fibrosis (F2–F4)			
	Univariate	P-value	Multivariate	P-value
Age	1.047 (1.022, 1.073)	<0.001		
Neutrophils	0.795 (0.651, 0.970)	0.024		
Lymphocytes	0.708 (0.468, 1.072)	0.103		
Monocytes	0.421 (0.056, 3.182)	0.402		
RBC	0.559 (0.339, 0.909)	0.019		
Hb	0.999 (0.986, 1.012)	0.832		
RDW-CV	1.014 (0.851, 1.208)	0.877		
RDW-SD	1.135 (1.063, 1.212)	<0.001	1.125 (1.048, 1.207)	0.001
PLT	0.989 (0.985, 0.994)	<0.001	0.990 (0.985, 0.995)	<0.001
TBIL	1.034 (1.001, 1.069)	0.045		
Albumin	0.965 (0.908, 1.025)	0.245		
Globulin	1.097 (1.038, 1.116)	0.001	1.084 (1.018, 1.155)	0.012
ALT	1.001 (0.999, 1.004)	0.303		
AST	1.005 (0.999, 1.012)	0.119		
ALP	1.02 (1.008, 1.032)	0.001	1.015 (1.002, 1.028)	0.021
GGT	1.005 (1.000, 1.009)	0.04		
LDH	1.003 (0.997, 1.009)	0.379		
CHE	1.000 (1.000, 1.000)	0.008		
INR	14.857 (1.554, 142.066)	0.019		
TT	1.271 (1.055, 1.531)	0.012		

RBC: red blood cell; Hb: hemoglobin; RDW-CV: the coefficient of variation of the red blood cell distribution width; RDW-SD: the standard deviation of the red blood cell distribution width; PLT: platelets; TBIL: total bilirubin; ALT: alanine aminotransferase; AST: aspartate aminotransferase; ALP: alkaline phosphatase; GGT: gamma-glutamyl transpeptidase; LDH: lactic dehydrogenase; CHE: cholinesterase; TT: thrombin time; INR: international normalized ratio.

### Comparisons of different noninvasive models according to the fibrosis stages

**Figure 2:**
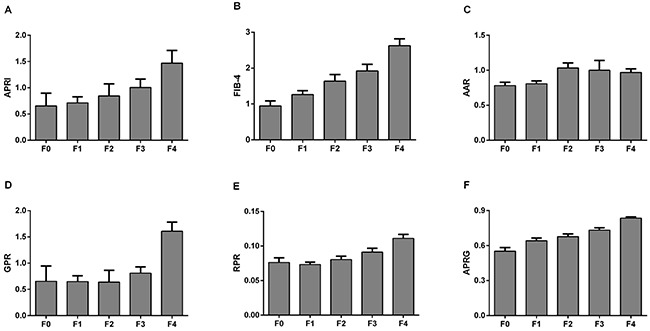
Comparisons of different noninvasive models according to the liver fibrosis stages in CHB patients

**Figure 3:**
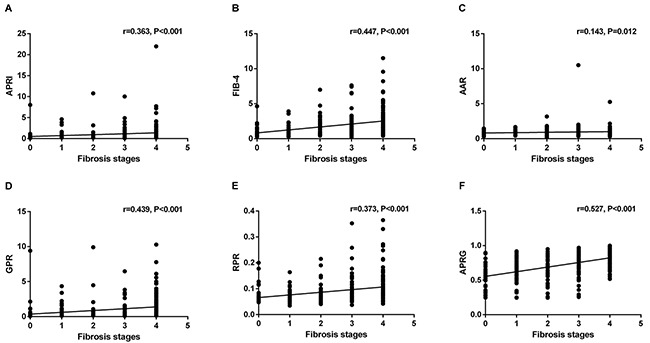
Correlations between different noninvasive models and liver fibrosis stages

Figure 2 presents the levels of APRI, FIB-4, AAR, GPR, RPR and the APRG index in the CHB patients according to liver fibrosis stages. These NITs showed an increasing trend with fibrosis stages in CHB patients. The correlations between NITs and liver fibrosis stages were analyzed by the Spearman's rank correlation coefficient analysis. Fibrosis stages were positively correlated with APRI (r=0.363, P<0.001), FIB-4 (r=0.447, P<0.001), AAR (r=0.143, P=0.012) and GPR (r=0.439, P<0.001), RPR (r=0.373, P<0.001) and the APRG index (r=0.527, P<0.001) (Figure 3).

### Comparisons of AUROCs between the APRG index and other established NITs

**Figure 4:**
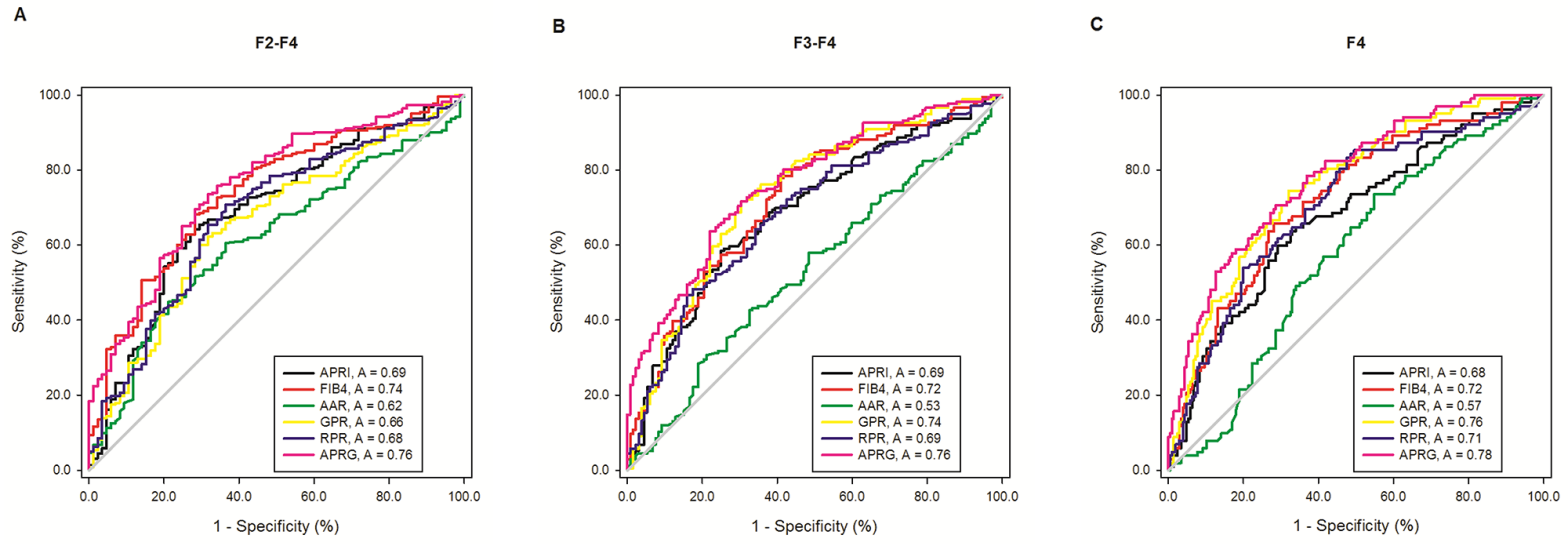
Receiver operating characteristic (ROC) curves of noninvasive blood biomarkers, including APRI, FIB-4, GPR, RPR, AAR and the APRG index level for significant liver fibrosis **(A)** advanced liver fibrosis **(B)** and liver cirrhosis **(C)** in the CHB patients.

Predictive values of the six models were evaluated by the receiver operating characteristic (ROC) curves for the entire CHB population according to their histological fibrosis stages (Table 3 and Figure 4). The areas under the ROC curves (AUROCs) of the APRG index in predicting significant fibrosis (≥F2), advanced fibrosis (≥F3) and liver cirrhosis (≥F4) were 0.757 (95%CI 0.699 to 0.816), 0.763 (95%CI 0.711 to 0.816) and 0.781 (95%CI 0.728 to 0.835), respectively. The optimal cut-off values of the APRG index for predicting significant fibrosis, advanced fibrosis and cirrhosis were 0.695, 0.777 and 0.787. For significant fibrosis, the AUROCs of the APRG index was significantly higher than APRI (0.692, 95%CI 0.626 to 0.758, P = 0.038), AAR (0.616, 95%CI 0.549 to 0.683, P = 0.002), GPR (0.657, 95%CI 0.590 to 0.724, P = 0.001) and RPR (0.681, 95%CI 0.614 to 0.747, P = 0.006), while there were no significant differences between the AUROCs of the APRG index and FIB-4 (0.738, 95%CI 0.678 to 0.799, P = 0.5). For predicting advanced fibrosis, the AUROCs of the APRG index was significantly better than APRI (0.689, 95%CI 0.630 to 0.749, P = 0.012), AAR (0.535, 95%CI 0.470 to 0.600, P < 0.001) and RPR (0.686, 95%CI 0.626 to 0.746, P = 0.002), but was comparable with FIB-4 (0.719, 95%CI 0.661 to 0.777, P = 0.093) and GPR (0.737, 95%CI 0.681 to 0.794, P = 0.383). For predicting cirrhosis, the APRG index exhibited a significantly higher AUROCs compared with APRI (0.676, 95%CI 0.612 to 0.740, P < 0.001), FIB-4 (0.723, 95%CI 0.663 to 0.783, P = 0.034), AAR (0.574, 95%CI 0.509 to 0.640, P < 0.001) and RPR (0.709, 95%CI 0.647 to 0.771, P = 0.009), while there were no significant differences between the AUROCs of the APRG index and GPR (0.758, 95%CI 0.702 to 0.814, P = 0.458).

**Table 3:** Diagnostic accuracy of different indexes for the prediction of liver fibrosis in the CHB patients

	Optimized cutoff	Sensitivity (%)	Specificity (%)	AUC (95%CI)	LR +	LR -	P value	P value of ROC contrast test*
**F0-F1 vs. F2-F4**								
APRI	0.544	65.47	70.59	0.692 (0.626, 0.758)	2.226	0.489	<0.001	0.038
FIB-4	1.205	68.16	71.76	0.738 (0.678, 0.799)	2.414	0.444	<0.001	0.500
AAR	0.802	60.54	63.53	0.616 (0.549, 0.683)	1.660	0.621	0.002	0.002
GPR	0.432	60.09	70.59	0.657 (0.590, 0.724)	2.043	0.565	<0.001	0.001
RPR	0.070	70.85	63.53	0.681 (0.614, 0.747)	1.943	0.459	<0.001	0.006
APRG	0.695	73.54	68.24	0.757 (0.699, 0.816)	2.316	0.388	<0.001	—
**F0-F2 vs. F3-F4**								
APRI	0.667	58.52	75.00	0.689 (0.630, 0.749)	2.341	0.553	<0.001	0.012
FIB-4	1.086	77.84	59.09	0.719 (0.661, 0.777)	1.903	0.375	<0.001	0.093
AAR	0.917	42.61	67.42	0.535 (0.470, 0.600)	1.308	0.851	0.295	<0.001
GPR	0.413	71.59	69.70	0.737 (0.681, 0.794)	2.363	0.408	<0.001	0.383
RPR	0.090	48.30	82.58	0.686 (0.626, 0.746)	2.773	0.626	<0.001	0.002
APRG	0.777	63.64	78.03	0.763 (0.711, 0.816)	2.897	0.466	<0.001	—
**F0-F3 vs. F4**								
APRI	0.667	64.71	66.02	0.676 (0.612, 0.740)	1.904	0.535	<0.001	<0.001
FIB-4	1.602	65.69	71.84	0.723 (0.663, 0.783)	2.333	0.478	<0.001	0.034
AAR	0.767	73.53	45.15	0.574 (0.509, 0.640)	1.341	0.586	0.034	<0.001
GPR	0.482	74.51	67.96	0.758 (0.702, 0.814)	2.326	0.375	<0.001	0.458
RPR	0.070	85.29	50.49	0.709 (0.647, 0.771)	1.723	0.291	<0.001	0.009
APRG	0.787	70.59	71.36	0.781 (0.728, 0.835)	2.465	0.412	<0.001	—

AUROC: area under the receiver operating characteristic curve; CI: confidence interval; LR-: negative likelihood ratio; LR+: positive likelihood ratio; AAR: aspartate aminotransferase to alanine aminotransferase ratio; APRI: aspartate aminotransferase to platelet ratio index; FIB-4: fibrosis index based on the four factors; GPR: gamma-glutamyl transpeptidase to platelet ratio; RPR: red cell distribution width to platelet ratio.

## DISCUSSION

Early detection and accurate assessment the severity of liver fibrosis is essential for antiviral therapy decisions in CHB patients [8]. Considering the limitations of LB, a simple predictive model for liver fibrosis using routinely serum-based biomarkers has always been an urgent assignment for clinician to avoid unnecessary LB. Although several non-invasive methods involving blood biomarkers such as APRI and FIB-4 to predict liver fibrosis have been developed over the past decade [13, 26], these methods were based on patients with CHC and the accuracy for assessing patients with CHB remains controversial. Recently, several models such as GPR, RPR and AAR have been used to assess the liver fibrosis of patients with CHB, but the accuracy and reliability of these indexes are not very satisfactory in predicting liver fibrosis [17, 27, 28].

In present study, we aimed to develop a novel inexpensive and routinely available model to predict significant fibrosis, advanced fibrosis and cirrhosis using blood-based parameters. Blood routine examination and biochemistry parameters, which are commonly tested during course of CHB patients, have essential implications for the natural history of chronic HBV infection. The PLT count is an independent risk factor in the most predictive models for liver fibrosis and cirrhosis. A large amount of studies reported that low PLT counts were associated with advanced liver fibrosis [29–32]. A possible explanation may be that the decreased PLT counts are due to splenomegaly and the decreased thrombopoietin production associated with liver cell failure in cirrhosis patients [33, 34]. RDW is a measure of the heterogeneity of the volume of red blood cells and is often used to diagnose different types of anemia. Recently, RDW has been demonstrated to be associated with cardiovascular and pulmonary diseases [35–37]. Felker et al. identified that high RDW was a strong independent predictor of outcome in patients with chronic heart failure [35]. Hampole et al. reported that RDW value was an independent predictor of mortality in patients with pulmonary hypertension [37]. RDW was also demonstrated as an independent predictor of the liver fibrosis stage in CHB patients. A retrospective study by Chen et al. showed that the RDW was a strong predictor of liver fibrosis in patients with CHB [28]. Lee et al. developed a novel index, the RPR, by using RDW and PLT. They found that RPR was comparable to APRI and FIB-4 but was inferior to TE for assessing significant fibrosis in a Korean population with chronic HBV infection [38]. However, it is worth noting that RDW can be expressed as RDW-SD and the coefficient of variation of the red blood cell distribution width (RDW-CV). Our findings indicated that RDW-CV was not a predictive risk factor for liver fibrosis. Instead, the RDW-SD was a strong and independent predictor of the liver fibrosis. RDW-CV is calculated from the erythrocyte volume distribution histogram. It represents the coefficient of variation of erythrocyte volume around mean corpuscular volume (MCV), while RDW-SD is determined from the width of erythrocyte volume distribution curve at level 20% above baseline and is expressed in femtoliters [39]. Caporal et al. repotted RDW-CV had higher sensitivity and efficiency in detecting anisocytosis in microcytic MCV ranges compared with RDW-SD. However, in normocytic and macrocytic MCV ranges, RDW-SD showed better efficiency in detecting anisocytosis than RDW-CV [40]. The reason why RDW-SD is associated with the stages of liver fibrosis is unclear and deserves further investigation. In addition, Schmilovitz-Weiss et al. and Xu et al. reported that there appears to be a strong association between serum level of globulin and extent of liver fibrosis in patients with chronic HBV infection [41, 42]. The correlation between globulin level and the degree of liver fibrosis was also found in our study through multivariate regression analysis. ALP is a hydrolase enzyme which is mainly expresses in the liver, bile duct, bone and so on [43]. In previous study, ALP has been also identified to be an independent predictor to liver fibrosis in CHB patients [44]. We found by forward stepwise multiple regression analysis, that ALP was also significantly correlated with different fibrosis stages. In the present study, other variables such as GGT, TBIL, AST, thrombin time (TT) and age were not the predictive factors for liver fibrosis. Conflicting findings are reported in previous studies on these serum parameters being independent variables determining the high fibrosis scores [16, 23, 30]. These discrepancies may due to the different study cohorts, which had liver disease caused by different pathogenic factors. Eventually, through multivariate analysis, RDW-SD, PLT, globulin and ALP were identified as the independent predictors of significant fibrosis in our study. Therefore, the APRG index was developed based on these four independent predictors of significant fibrosis.

The APRG index obtained higher AUCs than the APRI, FIB-4, RPR, GPR, AAR in the prediction of liver fibrosis. The APRG index is an accurately predictive index of significant fibrosis, advanced fibrosis and cirrhosis as compared with the APRI. Although WHO guideline suggests the use of APRI for estimating liver fibrosis in patients with CHB [8], many studies demonstrated that APRI and FIB-4 could identify liver fibrosis with an only moderate sensitivity and accuracy in CHB patients, and were not an ideal replacement for liver biopsy [14, 44, 45]. FIB-4 was first reported by Sterling et al. and was demonstrated to be useful for the diagnosis of liver fibrosis in CHC patients with co-infected HIV [23]. However, the diagnostic value of FIB-4 is still limited and controversial for chronically HBV-infected patients [16, 21]. In our study, although the diagnostic value of the APRG index was comparable with FIB-4 in identifying significant fibrosis and advanced fibrosis in CHB patients, the performance of the APRG index was slightly superior to that of FIB-4 in distinguishing liver cirrhosis. GPR is a newly noninvasive index to assess liver fibrosis in chronically HBV-infected patients and is superior to APRI and FIB-4 in West Africa cohorts [16]. However, Li et al. and Schiavon LL et al. found that GPR did not show any advantage in a Chinese cohort and a Brazilian cohort [17, 18]. In the present study, we found that as compared with the GPR, the APRG index had a more powerful predictive value for significant fibrosis but had equally powerful in the prediction of advanced fibrosis and cirrhosis. We assumed that the discrepancies in the diagnostic accuracy of GPR may be caused by laboratory methods, HBV genotypes and demographic characteristics. Although the GPR is a promising index for predicting liver fibrosis and includes only two inexpensive serum parameters, the value of GPR for distinguishing liver fibrosis in patients with CHB needs to be determined in further studies. Chen et al. reported that RPR is an accurately predictive index in identify significant fibrosis compared to APRI and FIB-4 [28]. However, Lee et al. verified that RPR has a similar performance with APRI and FIB-4 for assessing significant fibrosis in a Korean cohort of CHB [38]. Our study shown that RPR was inferior to the APRG index in identifying significant fibrosis, advanced fibrosis and cirrhosis. Other models, such as AAR was also proposed to assess the stages of liver fibrosis in patients with CHB recent years [27]. However, our study suggests that AAR is not a good method for the estimation of fibrosis stage compared with the APRG index.

The present study has several limitations. Firstly, our study is a retrospective study and the data was obtained from a single center. Secondly, our study did not consider the impact of the HBV genotypes, although the HBV genotypes of Asian are usually B or C [46]. Thirdly, since the parameters of serum HBV marker was insufficient, we did not consider the impact of these markers in liver fibrosis, and these factors should be considered in future studies. Fourthly, we did not compare the performance of APRG with TE for diagnosing liver fibrosis in CHB patients due to the absence of available data. Thus, whether the APRG is superior to TE for diagnosing liver fibrosis in CHB patients deserves further investigation.

## MATERIALS AND METHODS

### Patients

The present study included treatment-naïve patients who underwent LB at Huai’an No. 4 People's Hospital (Jiangsu, China) from 2008 to 2016. CHB is defined as having blood that tested positive for serum HBV surface antigen (HBsAg) > 6 months. Patients were excluded from this study for the following reasons: co-infection with hepatitis C virus (HCV), hepatitis D virus (HDV) and human immunodeficiency virus (HIV), primary biliary cirrhosis, autoimmune hepatitis, Wilson's disease, hepatocellular carcinoma (HCC) or other types of cancer, hematological diseases, acute heart failure and pregnancy.

All subjects provided written informed consent for the liver biopsy. The Ethics Committee of the Huai’an No. 4 People's Hospital, Jiangsu, China, approved the consent procedure and the study.

### Liver biopsy and laboratory test

LB was performed using 16-gauge biopsy needles under ultrasound guidance. To be considered as adequate for scoring, a minimum of 1 cm of liver tissue with at least five portal tracts was required. All the LB specimens were processed by formalin fixation, paraffin-embedding and stained with hematoxylin and eosin. All histological specimens were reviewed by pathologist blinded to patient clinical characteristics, according to the METAVIR scoring system [22]. Liver fibrosis was classified into the following five stages: F0, no fibrosis; F1, portal fibrosis without septa; F2, portal fibrosis with rare septa; F3, numerous septa without cirrhosis; and F4, cirrhosis [22]. Patient demographic and laboratory parameters were collected at the time of liver biopsy.

### Noninvasive prediction methods and calculation formulae

The noninvasive prediction methods and calculation formulae used in our study were as follows: APRI: (AST (U/L)/ULN of AST)/PLT count (10^9^/L) ×100 [11]; FIB-4: (age (years)×AST (U/L))/ ((PLT count (10^9^/L) × (ALT (U/L))^1/2^) [23]; AAR: AST (U/L)/ALT (U/L) [24]; GPR: (GGT (U/L)/ULN of GGT)/PLT count (10^9^/ L) ×100 [16]; RPR: RDW (%)/PLT count (10^9^/L) [25].

### Statistical analyses

The data analysis was performed using SPSS version 22.0 software (SPSS Inc., Chicago, IL, United States) and SigmaPlot version 12.5 (Systat Software Inc., San Jose, CA, United States). Continuous variables are expressed as median (interquartile range) and were compared using the Mann-Whitney U test. Categorical data were reported as percentages. Correlations were evaluated by the Spearman's rank correlation coefficient analysis. The risk factors for liver fibrosis in patients with CHB were analyzed by binary logistic regression. Variables having *P* values <0.05 in the univariate analysis were used for a multivariate stepwise logistic regression analysis. Binary logistic regression analyses were used to develop the predictive models of liver fibrosis and the final prediction model was selected using the forward stepwise procedures. Probabilities generated from the predictive models were recorded and used as new input variables for the ROC curve analysis. The diagnostic accuracy of serum model for liver fibrosis was evaluated by using the ROC curve. The AUROCs as well as 95% confidential interval (CI) of AUROC were calculated. Differences between the AUROCs were tested using the z-test. The cut-off values were determined by the Youden index which was the optimal combination of sensitivity and specificity. All P-value were 2-sided and any value of P < 0.05 was considered statistically significant.

## CONCLUSIONS

In summary, the present study demonstrated that PLT, RDW-SD, ALP and globulin were independent variables for determining the severity of liver fibrosis. The APRG index that is established using these four parameters has a better diagnosis performance compared to conventional blood biomarkers, such as APRI, FIB-4, GPR, RPR and AAR. Multi-center, prospective cohort studies are needed to confirm the diagnostic values of the APRG index and compare it with other established NITs for CHB patients in the future.

## Abbreviations

CHB: chronic hepatitis B
HBV: hepatitis B virus
CHC: chronic hepatitis C
HCV: hepatitis C virus
HDV: hepatitis D virus
HIV: human immunodeficiency virus
HCC: hepatocellular carcinoma
HBsAg: hepatitis B surface antigen
HBeAg: hepatitis B e antigen
LB: liver biopsy
TE: transient elastography
RBC: red blood cell
Hb: hemoglobin
RDW-CV: the coefficient of variation of the red blood cell distribution width
RDW-SD: the standard deviation of the red blood cell distribution width
PLT: platelets
MCV: mean corpuscular volume
TBIL: total bilirubin
ALT: alanine aminotransferase
AST: aspartate aminotransferase
ALP: alkaline phosphatase
GGT: gamma-glutamyl transpeptidase
LDH: lactic dehydrogenase
CHE: cholinesterase
TT: thrombin time
INR: international normalized ratio
AUROC: area under the receiver operating characteristic curve
CI: confidence interval
LR-: negative likelihood ratio
LR+: positive likelihood ratio
AAR: aspartate aminotransferase to alanine aminotransferase ratio
APRI: aspartate aminotransferase to platelet ratio index
FIB-4: fibrosis index based on the four factors
GPR: gamma-glutamyl transpeptidase to platelet ratio
RPR: red cell distribution width to platelet ratio
NITs: noninvasive fibrosis tests
